# Effect of a Novel E3 Probiotics Formula on the Gut Microbiome in Atopic Dermatitis Patients: A Pilot Study

**DOI:** 10.3390/biomedicines10112904

**Published:** 2022-11-11

**Authors:** Yiwei Wang, Chi Tung Choy, Yufeng Lin, Lin Wang, Jinpao Hou, Joseph Chi Ching Tsui, Junwei Zhou, Chi Ho Wong, Tai Ki Yim, Wai Kai Tsui, Un Kei Chan, Pui Ling Kella Siu, Steven King Fan Loo, Stephen Kwok Wing Tsui

**Affiliations:** 1 School of Biomedical Sciences, Faculty of Medicine, The Chinese University of Hong Kong, Hong Kong, China; 2 Microbiome Research Centre, BioMed Laboratory Company Limited, Hong Kong, China; 3 Department of Medicine and Therapeutics, Faculty of Medicine, The Chinese University of Hong Kong, Hong Kong, China; 4 Centre for Microbial Genomics and Proteomics, The Chinese University of Hong Kong, Hong Kong, China; 5 Hong Kong Institute of Integrative Medicine, Faculty of Medicine, The Chinese University of Hong Kong, Hong Kong, China; 6 Hong Kong Bioinformatics Centre, The Chinese University of Hong Kong, Hong Kong, China

**Keywords:** atopic dermatitis, gut microbiome, probiotic, metagenomics, machine learning, *Lactobacillus*

## Abstract

Atopic dermatitis (AD) has been shown to be closely related to gut dysbiosis mediated through the gut–skin axis, and thus the gut microbiome has recently been explored as a potential therapeutic target for the treatment of AD. Contrasting and varying efficacy have been reported since then. In order to investigate the determining factor of probiotics responsiveness in individuals with AD, we initiated the analysis of 41 AD patients with varying disease severity in Hong Kong, whereas the severity was assessed by Eczema Area and Severity Index (EASI) by board certified dermatologist. 16S rRNA sequencing on the fecal samples from AD patients were performed to obtain the metagenomics profile at baseline and after 8 weeks of oral administration of a novel E3 probiotics formula (including prebiotics, probiotics and postbiotics). While EASI of the participants were significantly lower after the probiotics treatment (*p* < 0.001, paired Wilcoxon signed rank), subjects with mild AD were found to be more likely to respond to the probiotics treatment. Species richness among responders regardless of disease severity were significantly increased (*p* < 0.001, paired Wilcoxon signed rank). Responders exhibited (1) elevated relative abundance of *Clostridium*, *Fecalibacterium*, *Lactobacillus*, *Romboutsia*, and *Streptococcus*, (2) reduced relative abundance of *Collinsella*, *Bifidobacterium*, *Fusicatenibacter*, and *Escherichia-Shigella* amid orally-intake probiotics identified using the machine learning algorithm and (3) gut microbiome composition and structure resembling healthy subjects after probiotics treatment. Here, we presented the gut microbiome dynamics in AD patients after the administration of the E3 probiotics formula and delineated the unique gut microbiome signatures in individuals with AD who were responding to the probiotics. These findings could guide the future development of probiotics use for AD management.

## 1. Introduction

Atopic dermatitis (AD) is a complicated chronic immune-mediated skin disorder presenting with remarkable and recurrent pruritic eczematous lesions, which could be provoked by environmental stimulus and skin hyperactivity. It could not only affect infants, children, and adolescents but also be increasingly identified in adults [1,2,3,4,5]. AD contributes as the leading cause of skin disorders globally and is one of the top non-fatal illnesses that may be regarded as an emerging endemic which poses a significant socio-economic burden [6].

Several factors are evidenced to be associated with AD incidence, susceptibility and severity, including but not limited to environmental factors, genetic composition, integrity of skin barrier, and variability in immune response. For example, it has been demonstrated that greater helper T cell Th17/Th22 polarization is observed in Asian populations with a combined manifest of AD and psoriasis [7,8]. Loss-of-function mutation in the filaggrin (*FLG*) gene is another well-recognized risk factor leading to severe AD with a diverge reported prevalence in different ethnic groups [9,10,11,12,13,14,15]. On top of the regular risk factors, a growing number of studies about the association between intestinal microbiome dysbiosis and AD has emerged owing to the recent advancement in next-generation sequencing (NGS) [13,16,17,18,19]. Decreased intestinal bacterial biodiversity, lower relative abundance of *Bifidobacterium*, *Akkermansia* and *Fecalibacterium*, depletion of *Coprococcus eutactus*, and enrichment of *Clostridia* and *Fecalibacterium prausnitzii* have been observed to be extremely related to the infants with eczema or AD onset early in life [17,20,21,22,23,24,25,26]. This association of gut microbiome with the skin condition is commonly known as the “gut-skin axis”, which was originally postulated in 1930 [27]. The concept and the importance of the axis are becoming increasingly appreciated by the wider dermatologists community nowadays [28].

Hence, the use of probiotics appeals as a possible intervention to augment the standard-of-care treatment of using moisturizing cream for hydration, topical/oral anti-inflammatory drugs for reducing inflammation. Compared with conventional therapies, probiotics have a favourable pharmacological profile and a low production cost, which makes it a more feasible and accessible option. At a cellular and molecular level, it has been demonstrated that probiotics could potentially regulate allergic responses through Th2 suppression and Treg activation [22,29,30,31,32]. There are a number of trials evaluating the clinical efficacy of the use of probiotics prenatally on mothers, infants, and children in preventing and treating AD, but the results remain inconclusive. Recent meta-analyses reckoned the administration of probiotics to significantly reduce SCORAD index in AD patients and might be beneficial in preventing AD onset with a less confident extent [10,16,24,25,29,31,32,33,34,35,36,37,38,39,40,41,42,43,44,45,46,47,48,49,50].

In this study, our group aims to evaluate the effectiveness and gut microbiome evolution upon the application of prebiotics, probiotics and postbiotics mixture in southern Chinese AD patients through 16S rRNA sequencing. The findings could help to evaluate, refine, and improve the clinical efficacy of probiotics as an intervention in AD patients.

## 2. Materials and Methods

**Study design** Forty-one adult (18–73 years) AD patients of Chinese ethnicity were recruited from a community trial through a collaboration between The Chinese University of Hong Kong and the BioMed Microbiome Research Centre. All participants (1) with chronic AD that has been present for at least 3 years before the screening visit with any severity; (2) aged above 18; and (3) who provided informed consent were included. Subjects with any one of the following conditions were not recruited or were excluded from the study: (1) history of adverse reaction to probiotics; (2) known overt bacterial infections in the skin; (3) known pregnancy; (4) premorbid medical conditions, such as cardiovascular, liver or renal dysfunction or diabetes mellitus; (5) having used oral corticosteroids, oral antibiotics, other immunosuppressive or any preparation of oral herbal medicines for the treatment of AD in the past one month; (6) having been diagnosed with scabies, allergic contact dermatitis, seborrheic dermatitis or psoriasis; and (7) had taken anti-coagulant or anti-platelet drugs in the past month. All forty-one recruited subjects were included in the subsequent analysis. All patients involved in this study were first diagnosed with AD and evaluated the AD severity by a professional dermatologist according to the EASI scale and fecal samples were collected for downstream sequencing. Then, the patients were orally administered the probiotic mixture for two consecutive months after which AD severity of each recruitment was assessed again and fecal samples were collected for the follow-up studies. Moreover, AD patients were separated into responders and non-responders according to the alterations of EASI score. Those whose AD severity score dropped by more than half before and after taking probiotic mixture were considered as responders, while non-responders were defined as the patients whose AD severity score increased, or did not decrease, or decreased by no more than half after probiotic mixture administration. Informed consent statements were obtained from all recruited subjects in this study. This study received approval from the Research Ethics Committee of Hong Kong Doctors Union. There was no change to the trial protocol after it commenced.

**Eczema Area and Severity Index** (**EASI**) EASI score assess the extent (i.e., area) and severity of inflamed areas in AD [51]. It covers 4 body regions, namely head and neck, trunk, upper limbs, and lower limbs. Each body region will be evaluated according to the average intensity of 4 signs including redness, thickness, scratching, and lichenification against a 3-point scale. The severity score of respective body region will be the sum of the average intensity score of the above-mentioned signs. Another component of EASI score involves the percentage of skin affected by AD rated against a 6-point scale. The final EASI score is the sum of severity score multiplied by area score and a multiplier of respective body region. EASI score could therefore range from 0 to 72 with higher score indicating worse severity [52]. Owing to the total number of subjects recruited, subjects were categorized into two subgroups of which subjects with EASI less than 16 were regarded as mild AD, and subjects with EASI larger than or equal to 16 were regarded as severe AD group.

**Probiotic mixture** All AD patients received daily capsule of a novel E3 probiotics formula developed by BioMed Microbiome Research Centre (BioMed Laboratory Company Limited, Hong Kong) containing a mixture of 7 types of highly effective gastro-resistant probiotics (not less than 2 × 10^10^ CFU/capsule at the time of production), effective postbiotic HK-LP (heat killed *L. plantarum*, 10 mg/capsule), and triple prebiotics containing inulin (22 mg/capsule), Galacto-oligosaccharides (GOS) (8.1 mg/capsule), and Fructo-oligosaccharides (FOS) (0.9 mg/capsule) for two months. The product was designed not as a single strain but as a bacteria mixture with *Lactobacilli* and *Bifidobacterium*. The probiotic mixture was composed of *Lactobacillus rhamnosus GG*, *Lactobacillus acidophilus GKA7*, *Lactococcus lactis GKL2*, *Lactobacillus casei GKC1*, *Lactobacillus paracasei GKS6*, *Bifidobacterium bifidum GKB2*, and *Bifidobacterium lactis GKK2*. *L. rhamnosus GG* formula was evidenced to reduce the occurrence and recurrence risks of allergy and eczema simultaneously and *B. lactis* was previously proved to strengthen the immunity system and improve symptoms of allergy and eczema [53,54,55,56,57]. Additionally, postbiotics HK-LP involved in this formula was proved to enhance the probiotics functions [58,59]. Moreover, prebiotics act as an energy source for probiotics, which not only enhance the probiotics function but also foster intestinal peristalsis as well as detoxification [60,61,62,63,64].

**Library Preparation and 16SrRNA** Sequencing All the fecal samples were processed in BioMed Laboratory (BioMed Laboratory Company Limited, Hong Kong) and were first homogenized in PurSafe^®^ DNA and RNA preservative (Puritan, Guilford, ME, USA) and subjected to beating with glass beads (425–600 μm, Sigma-Aldrich, Burlington, MA, USA) for 1 h by following the instructions provided. DNeasy Blood & Tissue Kit (Qiagen, Hilden, Germany) was used to conduct the isolation of Microbial DNA from fecal samples. The extracted DNA concentration of each sample was quantified using a Qubit™ dsDNA HS Assay Kit (Life Technologies, Carlsbad, CA, USA) with Qubit 3 Fluorometer (Thermo Fisher Scientific, Waltham, MA, USA). Amplicon library was constructed using 515F(5′-GTGCCAGCMGCCGCGG-3′)/907R(5′-CCGTCAATTTCMTTTRAGTTT-3′) primer pair spanning targeting at V4-V5 hypervariable of 16S rRNA genes, together with adapter sequences, multiplex identifier tags, and library keys. 16S rRNA gene sequencing was performed using the Illumina MiSeq platform (Illumina, Inc., San Diego, CA, USA) following the original Earth Microbiome Project Protocols. In the end, index barcodes and adapters removed pair-end clean reads were obtained for the downstream analysis [65].

**Microbiome bioinformatics analysis** Microbiome bioinformatics data were analyzed using a plugin-based system, QIIME 2-2021.4, integrating various microbiome analysis algorithms and tools [66]. Demultiplexed reads were firstly subjected to quality control and denoising filter of sequence data with DADA2 [67] using the q2-dada2 plugin to retrieve exact amplicon sequence variants (ASVs) [68]. All ASVs were then aligned with mafft [69] and then a phylogenetic tree was generated using fastree2 [70] via the q2-phylogeny plugin. Taxonomic annotation of the resulting ASV was carried out using the q2-feature-classifier [71] plugin and a pre-trained Naive Bayes classifier which was based on SILVA v138 taxonomic reference database with 99% similarity [72,73,74]. Diversity analyses were performed using the R package microeco (v0.3.2) [75]. We used six metrics to indicate alpha diversity: Observed OTUs, Chao1 Index (Chao1), ACE Index (ACE), Shannon Diversity Index (Shannon), Simpson Index (Simpson), and Faith’s phylogenetic diversity (PD). Furthermore, Beta diversity was calculated based on the Jaccard distance metric, Bray–Curtis distance metric, weighted UniFrac, and unweighted UniFrac distance metrics. The PERMANOVA test on beta diversity (999 permutations) was applied to compare the microbial community dissimilarity across groups using the adonis function in vegan R package to adjust the clinical variables and batch effects [76]. Differential abundance test between the Pre and Post groups was conducted using random forest and non-parametric test.

**Statistical analysis** All the statistical analysis and visualization of results were conducted in R 4.0.4. Shapiro–Wilk normality test were carried out for normality of all data. Demographic characteristics across groups were compared using Wilcoxon rank-sum tests for continuous variables and Chi-square tests or the Fisher exact test for categorical variables. Paired t-test or paired Wilcoxon signed-rank test was performed to determine the differences in AD severity and alpha diversity before and after probiotic use in the same patient. Statistical significance was set as a *p* < 0.05.

## 3. Results

### 3.1. Study Population

**Figure 1:**
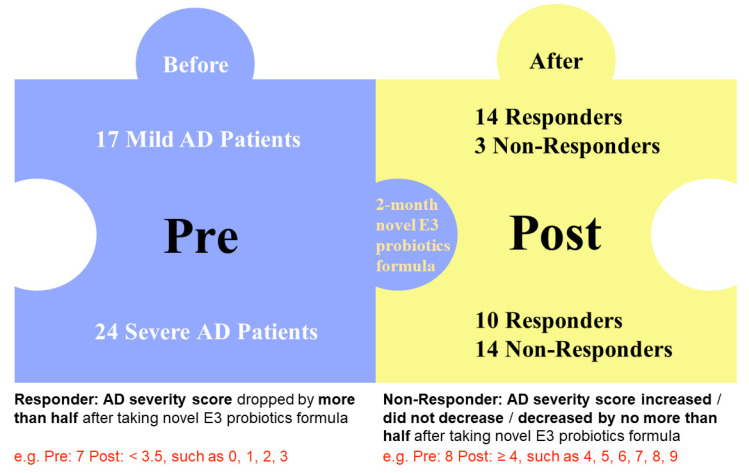
Study design.

A total of 41 AD patients were recruited in this study, including 17 mild AD patients and 24 severe AD patients. After 2 months of oral administration of probiotic mixture (one capsule daily), severity of AD patients was re-evaluated by a board certified dermatologist (S.K.F.L) with EASI. Significant improvement in AD severity was seen in 24 patients, which was considered as responders, 14 of which was from mild AD group and 10 was from severe AD group. The AD severity of the remaining 17 patients failed to improve, who were recognized as non-responders including 3 mild AD patients and 14 severe AD patients (Figure 1). As detailed in Table 1, the demographic characteristics and presence of comorbidity including sex (*p* = 0.5737), age (*p* = 0.8633), BMI (*p* = 0.3898), allergy (food allergy: *p* > 0.999 and other *p* = 0.7417, respectively), GI symptoms (constipation: *p* > 0.999 and diarrhea *p* = 0.2118) were similar between mild and severe AD subgroups. No other drugs were administrated during the study period.

### 3.2. Probiotic Mixture Significantly Ameliorates AD Severity

**Figure 2:**
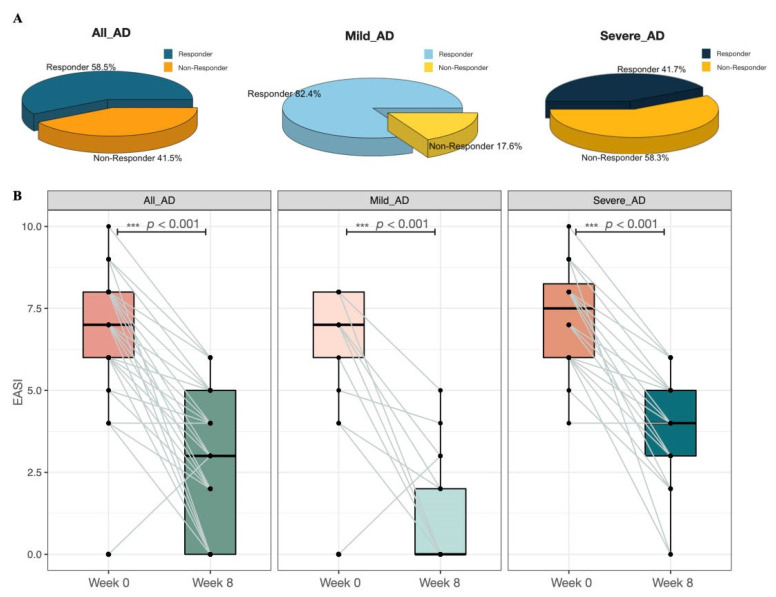
(**A**) Distribution of responders and non-responders among All_AD, Mild AD, and Severe AD patients. (**B**) Alteration of the AD severity score before and after novel E3 probiotics mixture administration among all AD, mild AD, and severe AD patients. *** denoted *p* < 0.001.

As shown in Figure 2, more mild AD patients significantly improved (*p* < 0.001) their AD condition after taking novel E3 probiotics formula, compared with the severe AD group. 82.4% of the patients in the mild AD group responded to the probiotics mixture, while only 41.7% of the patients in the severe AD group responded to the probiotic blend (Figure 2A). Our results also illustrated that the EASI of AD patients was significantly reduced (*p* < 0.001) after oral administration of the probiotic mixture regardless of baseline disease severity (Figure 2B).

### 3.3. Probiotic Mixture Improves the Diversity of Gut Microbiome in AD Patients

**Figure 3:**
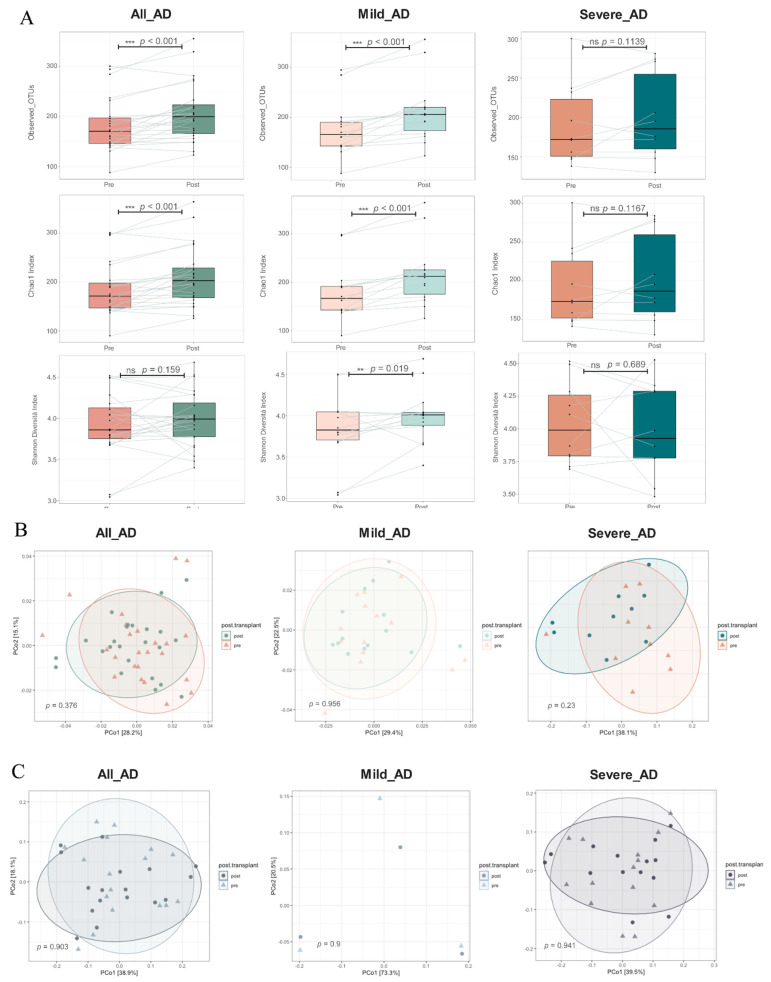
(**A**) Alteration of species richness, including three metrics, observed OTUs, Chao1 index and Shannon diversity index, before and after probiotic mixture intake among all AD, mild AD, and severe AD patients. (**B**) PCoA plots based on the weighted UniFrac distance metric across pre and post groups among the responders and (**C**) non-responders in All AD, Mild AD, and Severe AD patients. *p* value was calculated by PERMANOVA test with permutation = 999. ** denoted *p* < 0.05. *** denoted *p* < 0.001.

Alpha diversity, also called within-habitat diversity, is usually calculated to describe the richness and evenness of the community within a sample. The richness was measured by the Chao1 index, ACE index, and observed OTUs. Shannon diversity index and InvSimpson diversity index comprehensively consider the richness and uniformity of the community. For responders, significant increase of the species richness was obtained in mild AD patients (*p* < 0.001), while only a slight increase was identified in the severe AD group (*p* = 0.1139 for Observed OTUs; *p* = 0.1167 for Chao1 Index; Table S1, Figure 3A). In addition, for responders, a considerable increase of Shannon diversity index was obtained in mild AD group (*p* = 0.019) after taking probiotic mixture, while the Shannon diversity index of severe AD patients did not change significantly after the use of probiotics (*p* = 0.689; Table S1, Figure 3A). For non-responders, after probiotic mixture administration, no significant alteration was identified in alpha diversity of the gut microbiome among AD patients (Table S2, Figure S1). Moreover, in terms of beta diversity analysis, a similar intestinal bacterial community was obtained between pre and post groups in both responders and non-responders based on the Jaccard distance metric, Bray–Curtis distance metric, weighted UniFrac, and unweighted UniFrac distance metrics by PERMANOVA test (Tables S3 and S4, Figure 3B).

### 3.4. Gut Microbiome Profiling

**Figure 4:**
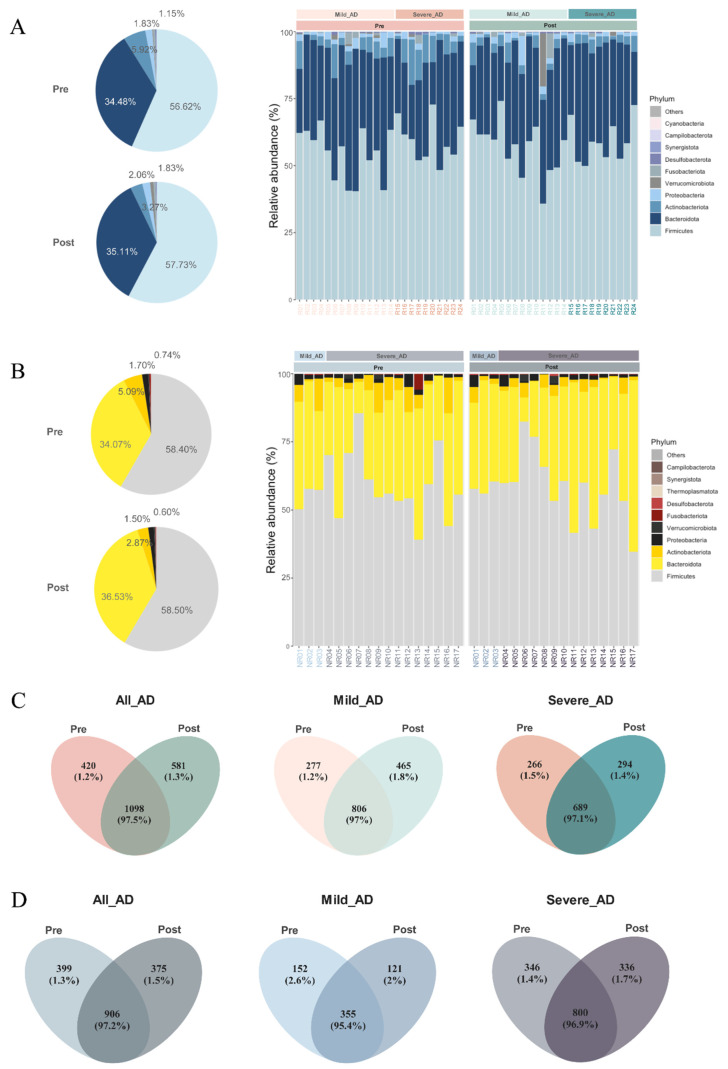
Gut microbiome composition of the pre and post groups at the phylum level for (**A**) responders and (**B**) non-responders. Venn diagrams illustrating the unique and shared ASVs of pre and post groups among (**C**) responders and (**D**) non-responders in All AD, Mild AD, and Severe AD groups. The integer data is ASV number. The percentage data is the sequence number/total sequence number.

At the phylum level, a total of 15 phyla, including 13 from the kingdom of bacteria and 2 from archaea, were detected in both responders and non-responders, and the top 4 most abundant phyla accounted for over 99% of sequences in the dataset. Before and after probiotic use, Firmicutes was dominant in the gut microbiome of all AD patients, followed by Bacteroidota, Actinobacteriota, and Proteobacteria (Figure 4A,B). At the genus level, the top five genera in the gut microbiome of responders were *Bacteroides*, *Fecalibacterium*, *Blautia*, *Bifidobacterium*, and *Fusicatenibacter*. However, in non-responders’ group, the top five genera were *Bacteroides*, *Blautia*, *Prevotella*, *Fecalibacterium*, and *Bifidobacterium* (Figures S2 and S3). After probiotics administration, 1098 ASVs were shared and persisted among responders. 420 unique ASVs and 581 unique ASVs were identified in pre and post groups separately (Figure 4C). For non-responders, Venn diagrams illustrated 399 and 375 unique ASVs from pre and post groups separately and a total of 906 shared ASVs (Figure 4D).

### 3.5. The Relative Abundance of Lactobacillus Increased Significantly after Oral Administration of Probiotic Mixture

**Figure 5:**
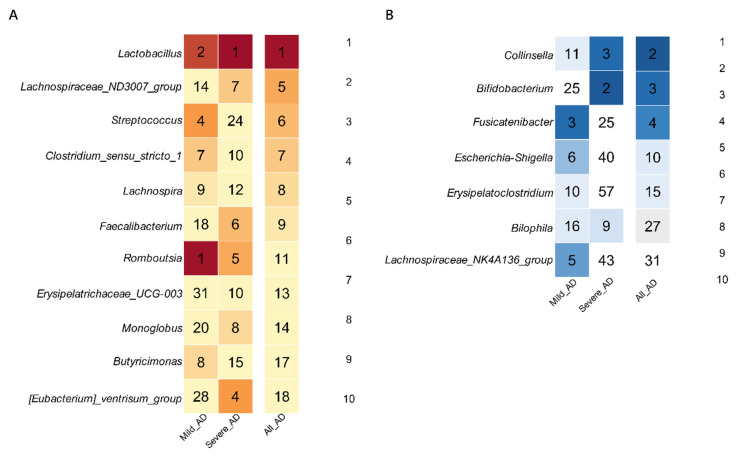
Significant genera selected across the pre and post groups in responders of (**A**) increasing and (**B**) decreasing trends. Only genera appearing in the ten top-ranking features in at least Mild AD or Severe AD groups were reported.

In order to determine the changes in gut microbiota composition following probiotic administration in AD patients, the random forest algorithm was conducted to select the key features affected by probiotics. Mean decrease in Gini coefficient was selected as the indicator value in the random forest analysis. The higher the value of the mean decrease in the Gini coefficient, the higher the importance of the genera responding to the treatment of probiotic mixture [77,78,79]. For responders, a total of 130, 98, and 92 features were identified between pre and post groups among all participants, mild AD and severe AD patients, respectively (Figure S4). We sorted the selected key features from high to low according to the mean decrease in the Gini coefficient and marked the rank of each genus in mild AD and severe AD group. We found that the relative abundance of *Lactobacillus*, *Lachnospiraceae_ND3007_group*, *Streptococcus*, *Clostridium_sensu_stricto_1*, *Lachnospira, Fecalibacterium*, *Romboutsia*, *Erysipelatrichaceae_UCG-003*, *Monoglobus*, *Butyricimonas*, and *[Eubacterium]_ventrisum_group* increased significantly after oral administration of probiotic mixture, while the relative abundance of *Collinsella*, *Bifidobacterium*, *Fusicatenibacter*, *Escherichia-Shigella*, *Erysipelatoclostridium*, *Bilophila*, and *Lachnospiraceae_NK4A136_group* decreased considerably (Figure 5). For non-responders, a total of 112, 25, and 99 features were identified between pre and post groups among all participants, Mild AD and Severe AD patients, respectively, and the relative abundance of *Lactobacillus* increased significantly (Figure S5).

## 4. Discussion

Despite the on-going debate on whether human gut microbiome dysbiosis is a cause or effect in the development of AD, there is convincing evidence showing that gut dysbiosis has a significant association with AD through the gut-skin axis. [16,47]. Therefore, probiotics have been explored as a therapeutic option for the treatment of AD [10,11,24,33,34,35,36,37,38,40,42,44,47,48,49,50]. In this study, we focus on the effect of probiotics in adult AD patients, the gut microbiome dynamics upon the course of probiotics and the gut microbiome signatures in responders under real world setting.

First of all, AD severity was significantly improved as evidenced by the drop in EASI after 8 weeks of oral probiotics in this cohort, although the minimal clinical important difference (MCID) had not been reached [80]. The effect of probiotics was more apparent in mild AD patients, likely because it would be relatively easier to restore the dysbiosis in mild AD patients than the heavily imbalanced gut flora in severe AD patients by probiotics [23]. Unsurprisingly, there is a surge in species richness among responders and it is consistent with the results reported by other groups [81]. For non-responders, no notable change in both alpha- and beta-diversity was observed. The taxonomic profile was highly comparable as illustrated in Figure 4 at the phylum level and in terms of ASVs across AD severity and time point.

At the genera level, our studies unrevealed the plausible colonization of probiotics in the responders’ gut. The probiotics strain, *Lactobacillus*, blended in the probiotics may directly colonize the gut microflora [81] or facilitate the expansion of existing beneficial communities. Further experiments would be required to validate the exact mechanism of colonization. Still, we presented concrete evidence that the successful colonization and/or expansion of *Lactobacillus* could be the key to stimulate response towards probiotics in AD patients. Furthermore, the relative abundances of commonly recognized beneficial bacteria including *Clostridium*, *Fecalibacterium*, *Romboutsia*, and *Streptococcus* were found to be enriched in AD patients after the course of probiotics, in addition to *Lactobacillus*. The expansion of beneficial bacteria could exert anti-inflammatory effects by the production of short-chain fatty acids (SCFAs) [20,82], including but not limited to acetate, butyrate, and propionate [83,84,85,86]. *Lachnospira* and *Butyricimonas* likely augment the production of SCFAs [87,88]. *Lachnospiraceae ND3007 group* and *[Eubacterium] ventrisum group* were also reported as a putative SCFA producer [88,89,90], while *Erysipelotrichaceae UCG-003* was reported to be closely related to *Fecalibacillus* genus [91], namely *Fecalibacillus intestinalis* and *Fecalibacillus faecis*. *Fecalibacillus intestinalis* and *Fecalibacillus faecis* are recently discovered bacterial species from human clinical samples [92] and is associated with Type II diabetes (T2D), hypertension and ageing [91,93]. Although the definitive role of both *Lachnospiraceae ND3007 group*, *[Eubacterium] ventrisum group*, and *Erysipelotrichaceae UCG-003* remains unclear, it is anticipated that they function similarly to modulate inflammation activity by SCFAs or other anti-inflammatory metabolites.

On the other hand, responders were characterized by the decline of relative abundance of detrimental bacteria (including *Collinsella*, *Escherichia-Shigella*) and other genera without an explicit role (*Fusicatenibacter*, *Erysipelatoclostridium* and *Bilophila*). For *Fusicatenibacter*, *Erysipelatoclostridium* and *Bilophila*, they have been described to correlate with a high fat diet, obesity, T2D, Crohn’s disease, and ulcerative colitis [94,95,96,97]. However, the role and relationship between the genera and AD are largely uncertain; it is anticipated that they would facilitate inflammation mediated by inducing the expression of pro-inflammatory cytokines, such as IL-17A [98]. Thus, lower relative abundance of these bacteria might relive the symptoms in AD patients. Nonetheless, the apparent reduction in the genera may not necessarily reflect the absolute bacteria counts [99]. Instead, the beneficial bacteria and SCFA-rich intestinal environment may outcompete these bacteria and discourage their expansion rather than inhibiting their growth.

Most importantly, the gut microbiome signatures among responders substantially overlapped with the gut microbiome signatures of AD patients previously reported by our group [100]. In particular, depletion of *Clostridium_sensu_stricto_1*, *Romboutsia*, and *Erysipelatrichaceae UCG-003* were detected in AD patients compared with healthy subjects, and their relative abundance were shown to be inversely correlated with AD severity. In this study, we reported the elevated relative abundance of both *Clostridium_sensu_stricto_1*, *Romboutsia*, and *Erysipelatrichaceae UCG-003* among responders with improving disease severity as evidenced by lower EASI score. An elevated relative abundance of *Erysipelatoclostridium* has been noted in AD patients, while the findings of reduced relative abundance of *Erysipelatoclostridium* in responders discussed herein further resonate the results. In other words, AD patients who responded to probiotics acquired a gut microbiome composition and structure resembling healthy subjects. To the best of our knowledge, this is the first depiction of gut microbiome composition shift from AD status to healthy status.

Interestingly, the relative abundance of *Lachnospiraceae NK4A136 group* and *Bifidobacterium* were significantly shrunken in the responders of mild and severe AD patients, respectively. The alterations could be an outcome instead of the causal driver of the responsiveness towards oral probiotics. For example, the *Bifidobacterium* in the probiotics blend and the pre-existing *Bifidobacterium* may compete for nutrients with each other and other bacteria in the gut, which *Lachnospiraceae NK4A136 group* may face comparable challenges in the presence of *Lachnospira*. Or in the contrary but less probably, the decrease in relative abundance might also reflect decrease shredding into stool following colonization. Nevertheless, it indicated a complicated reciprocity between micro-organisms in the human intestinal environment even though the observation might be counter-intuitive, and the utilization of probiotics to revert the gut microbiome balance from dysbiosis status might not be straightforward [101,102].

Taken all together, lines of evidence about the reshape of gut microbiome composition, especially in Southern Chinese atopic dermatitis patients in this study, has been presented. Although the duration of oral probiotics being taken, the optimal dosage of probiotics intake, and when should probiotics being administrated remain unresolved [101,102] due to the limitation of resources to conduct a more comprehensive longitudinal study, our findings hint at important clues on the effect of probiotics in AD patients and the distinctive microbiome signatures between responders and non-responders to probiotics. Reddel and colleagues conducted a 90-day gut microbiota profile with 3 time points recorded in 18 child AD patients from Italy [81], but further investigation to record the temporal evolution of gut microbiome composition and the persistence of probiotics in human gut upon the course of probiotics could potentially exacerbate the scientific ground for oral administration of probiotics for skin disease symptoms alleviation. Given the disparity of gut microbiome profile between mild and severe AD patients as previously reported [100], the impact of probiotics on the gut flora were expected to be inherently dissimilar, implying the potential of personalized probiotics blend [103,104] to improve efficacy with baseline and following microbiome profiling. Despite the fact that MCID could not be reached in this cohort, likely due to the relatively small sample size, it lays the foundation to incorporate probiotics into the management of AD patients. Of note, the role of fungi and virus in the gut flora of AD patients remains poorly elucidated. Shotgun metagenomics analysis could provide valuable insights in the whole picture down to the species and sub-species levels, with the hope that the complex interplay between numerous micro-organisms and hosts will be delineated in a more accurate and precise manner [105,106]. Last but not least, additional investigations would be required to validate and establish the definitive association of the above-mentioned observations and speculations with the management of AD in a generalizable manner.

## Figures and Tables

**Table 1:** Baseline Demographic and Disease Characteristics of Patients.

			Patients (No.)			
Variable			Overall (n = 41)	Mild AD (n = 17)	Severe AD (n = 24)	*p* Value
Characteristics						
	Sex, No. (%)					0.5737
		*Male*	16 (39.0)	8 (47.1)	8 (33.3)	
		*Female*	25 (61.0)	9 (52.9)	16 (66.6)	
	Age, mean (SD) [range], y		47.0 (15.6) [18–73]	47.6 (15.5) [26–66]	46.6 (16.0) [18–73]	0.8633
	Weight, mean (SD), kg		59.9 (11.1)	62.5 (11.1)	58.0 (10.9)	0.1414
	BMI, mean (SD) †		22.5 (3.3)	23.1 (3.3)	22.1 (3.3)	0.3898
	EASI, mean (SD)		17.7 (7.0)	10.7 (2.1)	22.7 (4.5)	<0.001
**Presence of Comorbidity**						
	Allergy ever, No. (%)					
		*Food allergy*	3 (7.3)	1 (5.9)	2 (8.3)	>0.999
		*Others*	14 (34.2)	5 (29.4)	9 (37.5)	0.7417
	GI, No. (%)					
		*Constipation*	14 (34.2)	6 (35.3)	8 (33.3)	>0.999
		*Diarrhea*	6 (14.6)	4 (23.5)	2 (8.3)	0.2118

BMI, body mass index; EASI, Eczema Area and Severity Index. † BMI between 23.0–25.0 kg/m^2^ is classified as overweight, while BMI > 25.0 kg/m^2^ is classified as obese.

## Data Availability

The raw sequence data are available in NCBI (BioProject PRJNA843860).

## Supplementary Materials

The following supporting information can be downloaded at: https://www.mdpi.com/article/10.3390/biomedicines10112904/s1, Figure S1 Alteration of Shannon diversity index before and after novel E3 probiotics formula intake among; Figure S2 Heatmap of the gut bacterial community of pre and post groups for responders at the genus level, only top 20 general in relative abundance was reported; Figure S3 Heatmap of the gut bacterial community of pre and post groups for non-responders at the genus level, only top 20 general in relative abundance was reported; Figure S4 Significant genera selected across the pre and post groups in responders of (A) All_AD patients, (B) Mild AD patients, and (C) Severe AD patients using the random forest algorithms with the parameters of tree number = 1000, boots = 30, nresam = 0.667. MeanDecreaseGini was used to indicate the importance of each genus. Only top 30 was displayed. Relative abundance of each genus in pre and post groups demonstrated on the right panel separately; Figure S5 Significant genera selected across the pre and post groups in non-responders of (A) All_AD patients, (B) Mild AD patients, and (C) Severe AD patients using the random forest algorithms with the parameters of tree number = 1000, boots = 30, nresam = 0.667. MeanDecreaseGini was used to indicate the importance of each genera. Only top 30 was displayed. Relative abundance of each genera in pre and post groups demonstrated on the right panel separately. Table S1 Alpha diversities among responders stratified by disease severity. Table S2 Alpha diversities among non-responders stratified by disease severity. Table S3 Beta diversities of responder stratified by disease severity. Table S4 Beta diversities of non-responders stratified by diseases severity.
