# Supplementary material for: Effect of a Novel E3 Probiotics Formula on the Gut Microbiome in Atopic Dermatitis Patients: A Pilot Study
Source: Biomedicines. 2022 Nov 11;10(11):2904. doi: 10.3390/biomedicines10112904 (PMC9687608; doi:10.3390/biomedicines10112904)
Supplement: Supplementary file 1 [file biomedicines-10-02904-s001.zip › TableS1.pdf]

Measure	Responders: Pre VS Post								
	ALL_AD (n=24)			Mild_AD (n=14)			Severe_AD (n=10)		
	trend(post-pre)	*p* value	Sig.	trend(post-pre)	*p* value	Sig.	trend(post-pre)	*p* value	Sig.
**Observed_OTUs**	↑	0.0001	***	↑	8.5E-05	***	↑	0.114	
**Chao1**	↑	9.4e-05	***	↑	5.7e-05	***	↑	0.117	
**ACE**	↑	0.0002	***	↑	9.1e-05	***	↑	0.108	
**Shannon**	↑	0.159		↑	0.019	**	↓	0.089	
**Simpson**	↑	0.330		↑	0.140		↓	0.728	
**InvSimpson**	↑	0.551		↑	0.271		↓	0.695	
**Fisher**	↑	0.551		↑	0.0001	***	↑	0.113	
**Coverage**	↑	0.996		↑	0.989		↓	0.894	
**PD**	↑	0.211		↑	0.035	**	↑	0.191	
