# Supplementary material for: Effect of a Novel E3 Probiotics Formula on the Gut Microbiome in Atopic Dermatitis Patients: A Pilot Study
Source: Biomedicines. 2022 Nov 11;10(11):2904. doi: 10.3390/biomedicines10112904 (PMC9687608; doi:10.3390/biomedicines10112904)
Supplement: Supplementary file 1 [file biomedicines-10-02904-s001.zip › TableS2.pdf]

Measure	Non-Responders: Pre VS Post								
	ALL_AD (n=17)			Mild_AD (n=3)			Severe_AD (n=14)		
	trend(post-pre)	*p* value	Sig.	trend(post-pre)	*p* value	Sig.	trend(post-pre)	*p* value	Sig.
**Observed_OTUs**	↑	0.670		↓	0.766		↑	0.451	
**Chao1**	↑	0.678		↓	0.737		↑	0.542	
**ACE**	↑	0.644		↓	0.756		↑	0.426	
**Shannon**	↓	0.729		—	0.496		↓	0.732	
**Simpson**	↓	0.875		—	1		↓	0.894	
**InvSimpson**	↓	0.649		↓	0.619		↓	0.611	
**Fisher**	↑	0.644		↓	0.421		↑	0.426	
**Coverage**	—	0.754		—	1		—	0.724	
**PD**	↓	0.680		↓	0.167		↓	0.591	
