# Supplementary material for: Effect of a Novel E3 Probiotics Formula on the Gut Microbiome in Atopic Dermatitis Patients: A Pilot Study
Source: Biomedicines. 2022 Nov 11;10(11):2904. doi: 10.3390/biomedicines10112904 (PMC9687608; doi:10.3390/biomedicines10112904)
Supplement: Supplementary file 1 [file biomedicines-10-02904-s001.zip › TableS3.pdf]

Type	Group	Metric	*p* value	Sig.
Responder	All_AD: Pre VS Post	Jaccard distance metric	0.995	
		Bray-Curtis distance metric	0.997	
		Unweighted UniFrac distance metric	0.742	
		Weighted UniFrac distance metric	0.376	
	Mild_AD: Pre VS Post	Jaccard distance metric	0.993	
		Bray-Curtis distance metric	1	
		Unweighted UniFrac distance metric	0.606	
		Weighted UniFrac distance metric	0.956	
	Severe_AD: Pre VS Post	Jaccard distance metric	0.971	
		Bray-Curtis distance metric	0.992	
		Unweighted UniFrac distance metric	0.974	
		Weighted UniFrac distance metric	0.230	
