# Supplementary material for: Effect of a Novel E3 Probiotics Formula on the Gut Microbiome in Atopic Dermatitis Patients: A Pilot Study
Source: Biomedicines. 2022 Nov 11;10(11):2904. doi: 10.3390/biomedicines10112904 (PMC9687608; doi:10.3390/biomedicines10112904)
Supplement: Supplementary file 1 [file biomedicines-10-02904-s001.zip › TableS4.pdf]

Type	Group	Metric	*p* value	Sig.
Non-Responder	All_AD: Pre VS Post	Jaccard distance metric	0.999	
		Bray-Curtis distance metric	0.999	
		Unweighted UniFrac distance metric	0.962	
		Weighted UniFrac distance metric	0.903	
	Mild_AD: Pre VS Post	Jaccard distance metric	0.717	
		Bray-Curtis distance metric	1	
		Unweighted UniFrac distance metric	0.778	
		Weighted UniFrac distance metric	0.9	
	Severe_AD: Pre VS Post	Jaccard distance metric	0.999	
		Bray-Curtis distance metric	0.999	
		Unweighted UniFrac distance metric	0.963	
		Weighted UniFrac distance metric	0.941	
