# Supplementary figures and images for: Effect of a Novel E3 Probiotics Formula on the Gut Microbiome in Atopic Dermatitis Patients: A Pilot Study
Source: Biomedicines. 2022 Nov 11;10(11):2904. doi: 10.3390/biomedicines10112904 (PMC9687608; doi:10.3390/biomedicines10112904)

A

All\_AD

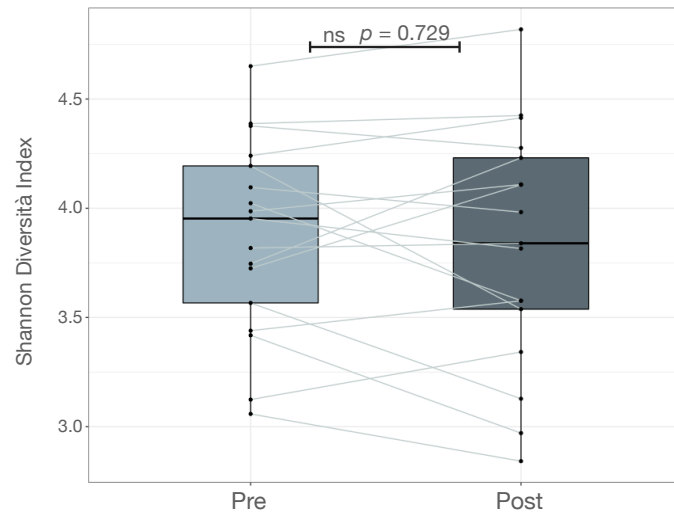

B

Mild\_AD

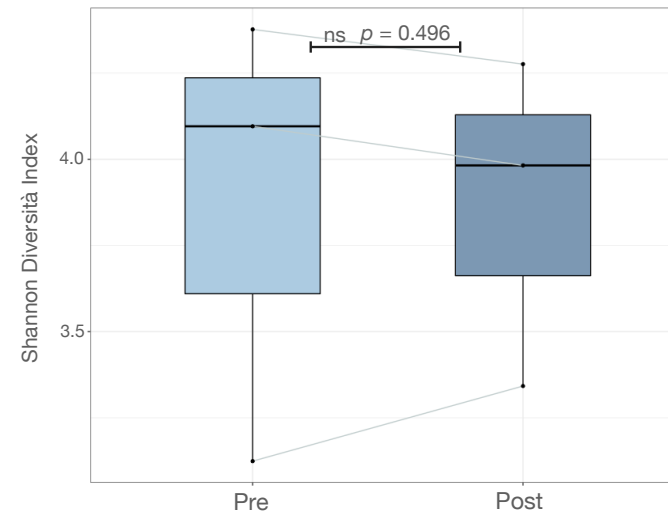

C

Severe\_AD

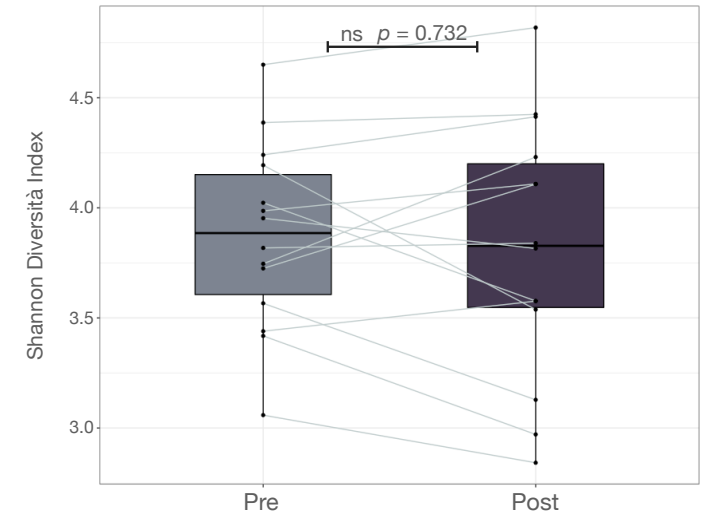

Supplement: Supplementary file 1 [file biomedicines-10-02904-s001.zip › FigureS1.pdf]

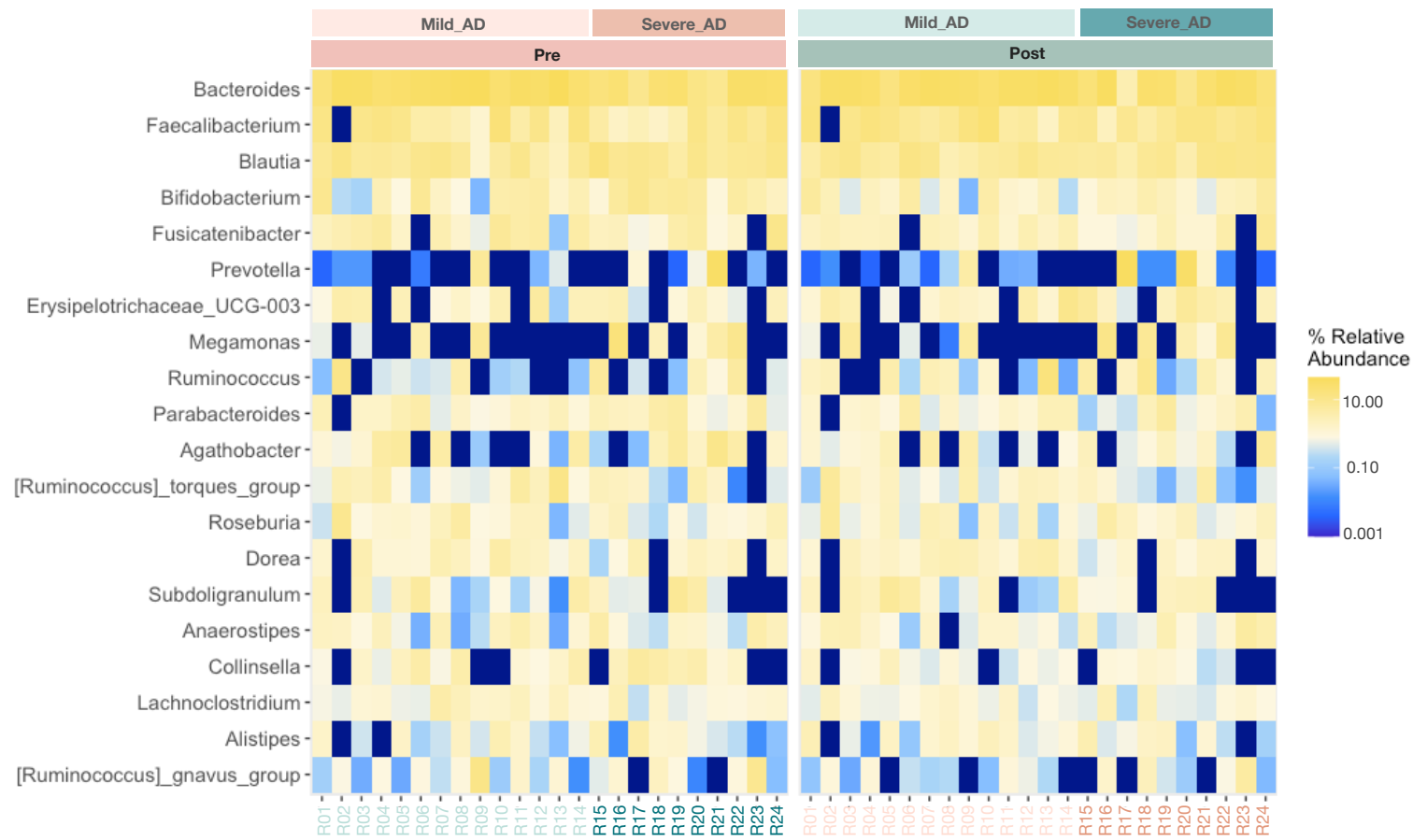

Supplement: Supplementary file 1 [file biomedicines-10-02904-s001.zip › FigureS2.pdf]

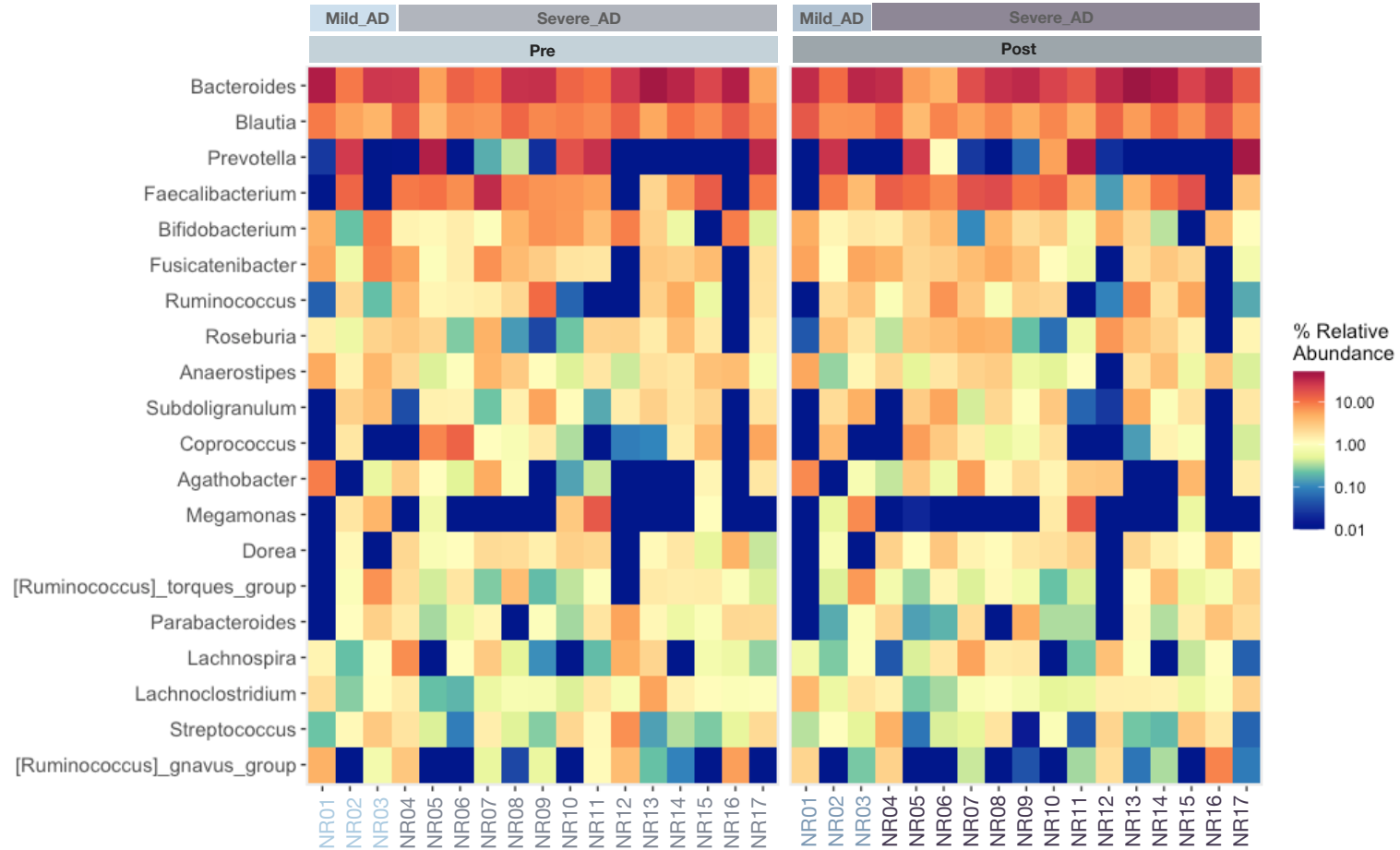

Supplement: Supplementary file 1 [file biomedicines-10-02904-s001.zip › FigureS3.pdf]

A

All\_AD

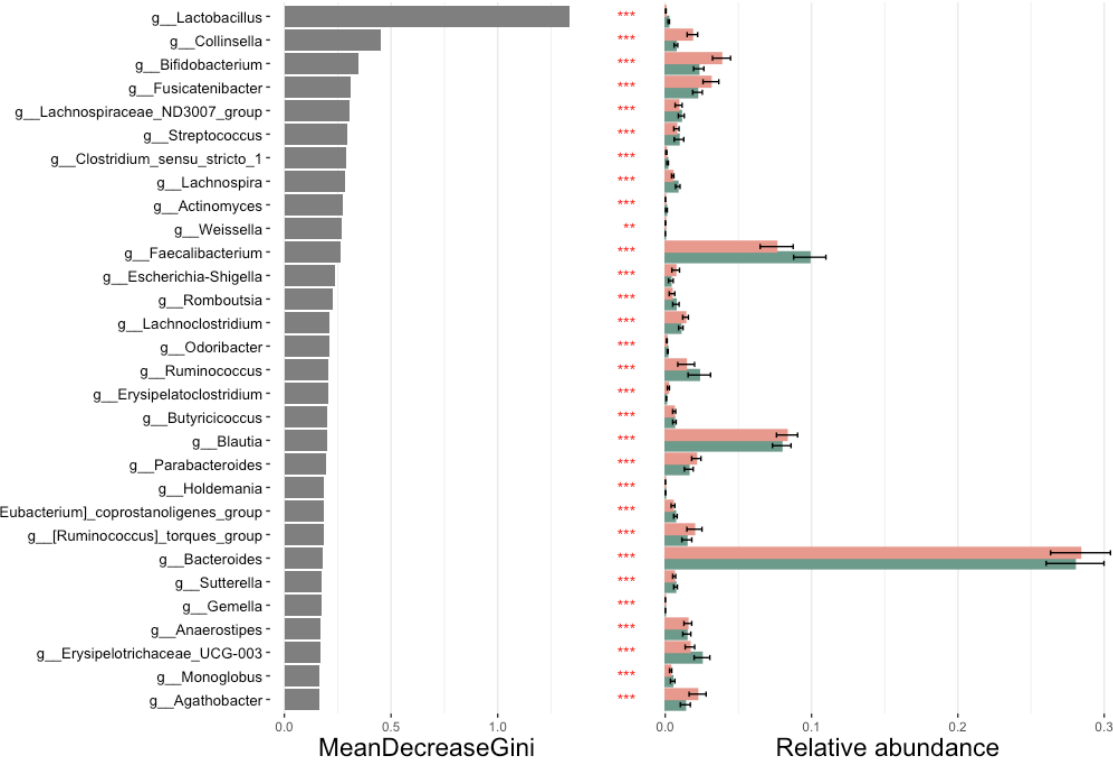

B

Mild\_AD

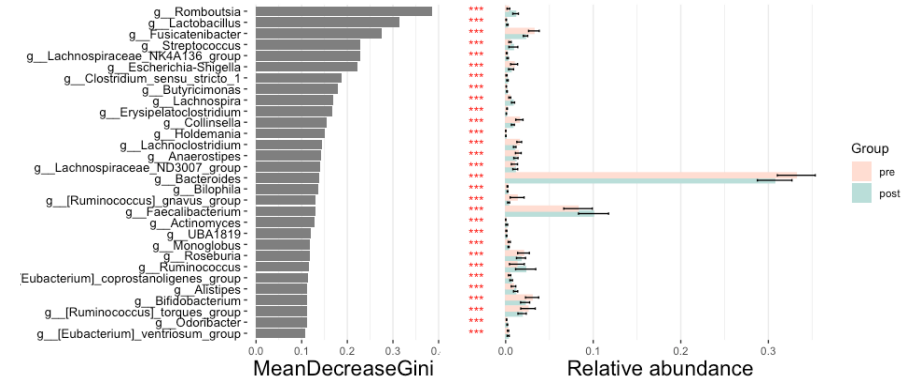

C

Severe\_AD

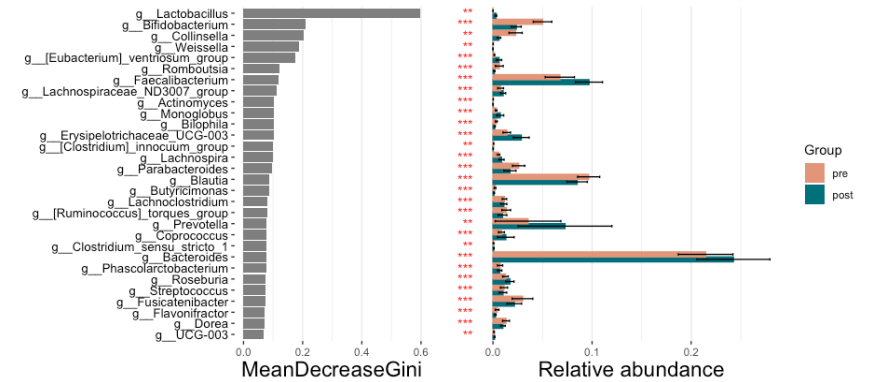

Supplement: Supplementary file 1 [file biomedicines-10-02904-s001.zip › FigureS4.pdf]

A

All\_AD

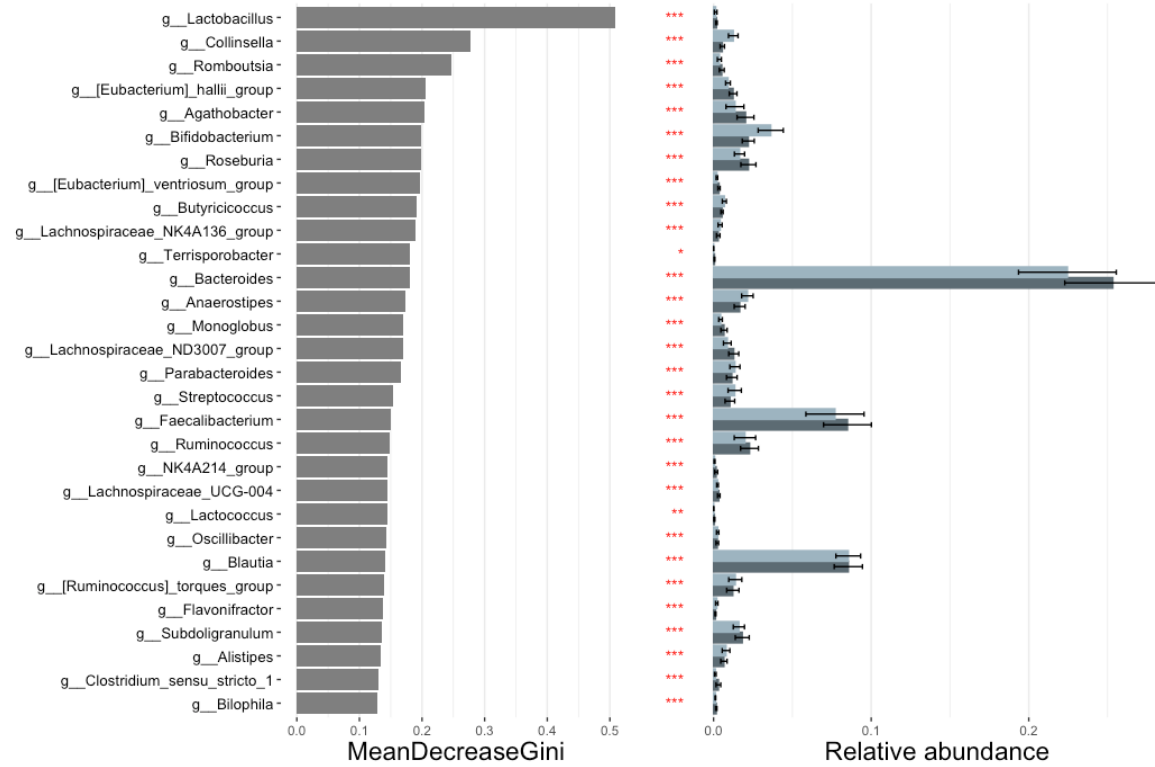

B

Mild\_AD

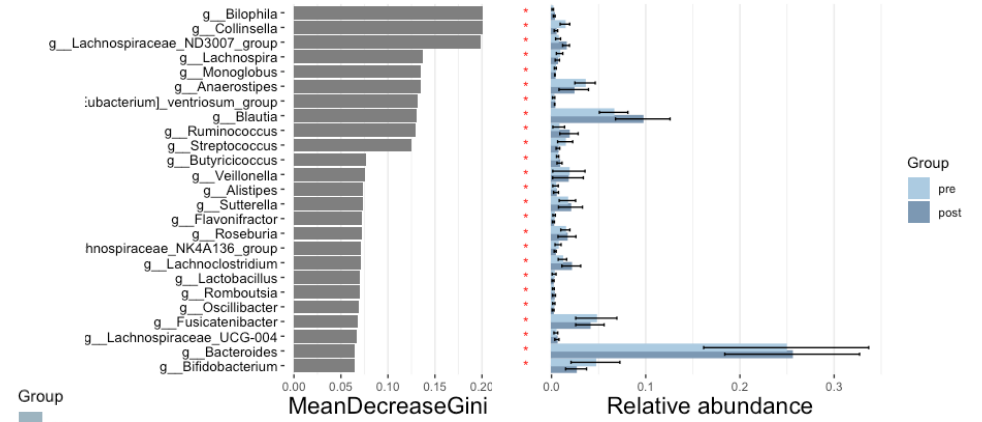Group  
pre  
post

C

Severe\_AD

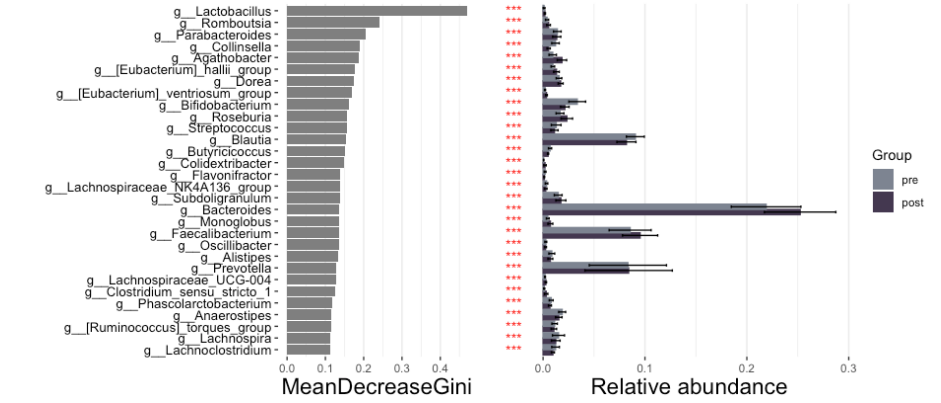Group  
pre  
post

Supplement: Supplementary file 1 [file biomedicines-10-02904-s001.zip › FigureS5.pdf]
